# Rising XDR-Typhoid Fever Cases in Pakistan: Are We Heading Back to the Pre-antibiotic Era?

**DOI:** 10.3389/fpubh.2021.794868

**Published:** 2022-01-17

**Authors:** Muhammad Hammad Butt, Aqsa Saleem, Syed Owais Javed, Irfan Ullah, Mujeeb Ur Rehman, Nayyer Islam, Muhammad Azam Tahir, Tangina Malik, Sara Hafeez, Shahzadi Misbah

**Affiliations:** ^1^Faculty of Pharmacy, University of Central Punjab, Lahore, Pakistan; ^2^Dow University of Health Sciences, Karachi, Pakistan; ^3^Kabir Medical College, Gandhara University, Peshawar, Pakistan; ^4^Department of Pharmaceutics, Faculty of Pharmaceutical Sciences, Government College University, Faisalabad, Pakistan; ^5^Khaldunia college of Pharmacy, Lahore, Pakistan; ^6^University of the Punjab, Lahore, Pakistan; ^7^Department of Biotechnology, Quaid-i-Azam University, Islamabad, Pakistan

**Keywords:** infectious disease, outbreak, COVID-19, Pakistan, tropical disease

The world witnessed its first case of Extensively Drug-Resistant—Typhoid Fever (XDR-TF) in late 2016 when Pakistan's second most populated province—Sindh—reported a huge influx of blood-culture confirmed Typhoid Fever (TF) cases that were refractory to standard therapy (1). Since then, according to the Weekly Field Epidemiological Report by the National Institute of Health (NIH) Islamabad, a total of 14,360 XDR-TF has been reported in Karachi from January 2017 till June 2021, and from November 2016 to June 2021 a total of 5,741 confirmed cases of XDR-TF were reported in all districts of Sindh province (excluding Karachi), while 69.5% cases were reported from District Hyderabad (2). In 2019, Pakistan became the first country in the world to introduce the World Health Organization (WHO)-recommended typhoid conjugate vaccine (TCV) into its routine immunization program (1).

Typhoid fever, caused by the bacterium *Salmonella typhi*, is characterized by clinical features such as step-ladder patterned high-grade fever, constipation or diarrhea, chills, and myalgias, etc. The microbe has a fecal-oral route of transmission, and it primarily spreads in low-resource areas with a poor socioeconomic standing. Lack of proper sanitation facilities, use of contaminated water, and neglect toward food safety guidelines are all common ingredients that make up the perfect breeding ground for this organism to thrive in (3).

Globally, around 21 million people are affected by this bacterium, with almost 161,000 deaths reported, each year. While the introduction of antibiotics has limited the prevalence of typhoid fever, to our dismay, the causative agent has developed resistance to multiple drugs over the years via different mechanisms. This has given birth to newer and more powerful strains, such as Multi-Drug Resistant (MDR)-Typhoid: resistant to three antibiotics; ampicillin, trimethoprim-sulfamethoxazole, and chloramphenicol, and XDR-Typhoid: resistant to five antibiotics; chloramphenicol, ampicillin, co-trimoxazole, fluoroquinolones, and third-generation cephalosporins. Only three antimicrobial drugs, namely: azithromycin (oral), carbapenems, and tigecycline (parenteral) are effective against the XDR strains (1). This, in addition to restricting treatment options for physicians, also poses an increased threat to patients who might develop a severe life-threatening illness.

Pakistan has been in an ongoing struggle against XDR-TF for almost 5 years now. There has been a recent surge in cases observed in its most populated city, Karachi. According to the Pakistan National Institute of Health, the city witnessed 52 new cases of XDR-Typhoid fever in the week preceding August 14, bringing the total cases reported between January 01, 2017, and Aug 14, 2021, to 15,224 (3). This sudden rise in drug-resistant cases is not only alarming for the country, but it is also a matter of grave concern for healthcare systems worldwide, due to fear of international outbreaks.

While the lack of proper sanitation and indiscriminate prescription of antibiotics remains to be a predominant cause in the spread of typhoid in low—and middle-income countries like Pakistan, the recent increase in cases suggests that there are other problematic factors at play that need to be addressed. One common reason implicated to be involved in the spread of typhoid in these regions is the use of contaminated water for drinking and irrigation. Additionally, amid the global pandemic COVID-19, which emerged in December 2019 and has been the cause of tremendous panic around the globe due to its subsequent emerging waves, (4) researchers have pointed out the impact of complications related to COVID-19 that the public has to face, for instance, heart-related diseases, pneumothorax, acute respiratory distress syndrome, and secondary infections (5) that can lead to co-epidemics and co-infections of COVID-19 and other infectious diseases putting more burden on already overburdened healthcare infrastructure (6, 7). During the current pandemic, poor sanitation is causing a surge in typhoid and the existing outdated diagnostic methods are paving the way toward irrational pharmacotherapy. Particularly, research has highlighted the overuse of azithromycin to treat COVID-19 and this practice might impair one of the few remaining solutions against the XDR *S. typhi*. Facing COVID-19 and XDR simultaneously can result in a catastrophe in Pakistan because it is entirely occupied with the pandemic and it has been reported that around 20,000 typhoid cases within a period of 10 days during June 2020 have been diagnosed along with COVID-19 (8). Such an unfortunate situation can cause havoc and it would heavily affect the already compromised public health sector that is losing due to the inability to serve and care for all the patients at the same time with limited financing and healthcare resources (7). A study conducted by M.K Daud et al. showed that water samples acquired from Karachi—the economic hub of Pakistan—had evidence of microbial contaminants (9). This highlights that the water available for consumption by the masses is not only unhealthy but also a live source of disease propagation. Another possible reason for the increasing typhoid cases could be the rise in the urban population of big cities, such as Karachi, as people from underdeveloped areas migrate here in search of business and/or refuge. According to recent statistics, the population of Karachi has grown to around 16 million and is expected to rise over 23 million by 2035 (10). This rapidly increasing population means that a large proportion has to live in slums and overcrowded areas, where basic life utilities like the proper sewage system, availability of potable water, and access to clean food are simply beyond reach. To make things worse, people living in these areas lack basic awareness regarding good hygiene practices and the spread of infectious diseases, making them more vulnerable to the plight of typhoid fever. Moreover, inhabitants of these areas do not have easy access to affordable and quality healthcare facilities. And, lack of competent medical personnel working in such facilities further contributes to the worsening of the prevailing antimicrobial resistance. In addition to this, cultural and religious barriers toward vaccines have played a key role in preventing widespread vaccination against typhoid which is the most effective tool to date for limiting the spread of the disease (11).

All things considered; it can rightly be assumed that the control of the XDR strain has become a challenge for healthcare providers across the country. Out of the three drugs commonly used against XDR TF, azithromycin remains to be the only available option that is administered orally, thus being the last resort of treating people in an out-patient setting. Recent studies, however, have shown that the XDR typhoid is now growing more insusceptible to the use of azithromycin. According to a recent research, 57 cases of blood culture-proven patients of Salmonella typhi at CMH Lahore, Punjab were reported during January 2019 to August 2020, out of which only 10 cases were nonresistant, seven were confirmed as Multi Drug-Resistant (MDR) and worryingly, 39 among these cases were Extensively Drug-Resistant (XDR). An even bigger matter of concern was that the *S. typhi* isolated from one of these cases was also resistant to azithromycin along with other first-line drugs (12). This unfortunate situation is subsequently forcing physicians to use carbapenems and tigecycline, both of which are administered parenterally. These drugs are expensive, and also relatively inaccessible for the poverty-stricken populace of developing countries since they require in-patient management. Moreover, policymakers of several countries have expressed concerns over the impending global burden of XDR typhoid outbreaks, as cases begin to rise at an alarming rate. Therefore, as a measure of the counter, the new CDC guidelines recommend all passengers traveling to this part of the world get vaccinated against typhoid (13).

In conclusion, while vaccination is an effective, short-term solution to curb the spread of XDR TF, a holistic approach entailing sustainable long-term measures needs to be implemented. Provision of adequate sanitation, clean drinking water, and a system for waste management should be ensured by the government in underprivileged areas, as they are the primary source of transmission of infectious diseases. Additionally, widespread vaccination programs and public awareness campaigns addressing proper hygiene practices should be routinely conducted. Knowing that antimicrobial resistance leads to the emergence of newer more resistant strains, strict laws should be set in place to ensure that healthcare workers do not over-prescribe antibiotics.

## Author Contributions

MB, AS, SJ, and IU : substantial contributions to the conception or design of the work or the acquisition and analysis or interpretation of data for the work. AS, MR, NI, MT, TM, SH, and SM: drafting the work. MB and IU: revising the manuscript critically for important intellectual content. MB: accountable for all aspects of the work in ensuring that questions related to the accuracy or integrity of any part of the work are appropriately investigated and resolved. All authors contributed to the article and approved the submitted version.

## Conflict of Interest

The authors declare that the research was conducted in the absence of any commercial or financial relationships that could be construed as a potential conflict of interest.

## Publisher's Note

All claims expressed in this article are solely those of the authors and do not necessarily represent those of their affiliated organizations, or those of the publisher, the editors and the reviewers. Any product that may be evaluated in this article, or claim that may be made by its manufacturer, is not guaranteed or endorsed by the publisher.
